# Effects of Hinokitiol and Dicalcium Phosphate on the Osteoconduction and Antibacterial Activity of Gelatin-Hyaluronic Acid Crosslinked Hydrogel Membrane In Vitro

**DOI:** 10.3390/ph14080802

**Published:** 2021-08-16

**Authors:** Kai-Chi Chang, Wen-Cheng Chen, Ssu-Meng Haung, Shih-Ming Liu, Chih-Lung Lin

**Affiliations:** 1 Advanced Medical Devices and Composites Laboratory, Department of Fiber and Composite Materials, Feng Chia University, Taichung 407, Taiwan; ketty60221@gmail.com (K.-C.C.); dream161619192020@gmail.com (S.-M.H.); 0203home@gmail.com (S.-M.L.); 2 Department of Fragrance and Cosmetic Science, College of Pharmacy, Kaohsiung Medical University, Kaohsiung 807, Taiwan; 3 Division of Neurosurgery, Department of Surgery, Kaohsiung Medical University Hospital, Kaohsiung 807, Taiwan; 4 Department of Surgery, School of Medicine, College of Medicine, Kaohsiung Medical University, Kaohsiung 807, Taiwan; 5 Graduate Institute of Medicine, College of Medicine, Kaohsiung Medical University, Kaohsiung 807, Taiwan

**Keywords:** hydrogels, membranes, dicalcium phosphate, osteoconductive, antibacterial

## Abstract

Many hydrogel-based crosslinking membranes have been designed and tailored to meet the needs of different applications. The aim of this research is to design a bifunctional hydrogel membrane with antibacterial and osteoconducting properties to guide different tissues. The membrane uses gelatin and hyaluronic acid as the main structure, 1-(3-dimethylaminopropyl)-3-ethylcarbodiimide hydrochloride as the crosslinker, hinokitiol as the antibacterial agent, and dicalcium phosphate anhydrous (DCPA) micron particles for osteoconduction. Results show that the hydrogel membrane with added DCPA and impregnated hinokitiol has a fixation index higher than 88%. When only a small amount of DCPA is added, the tensile strength does not decrease significantly. The tensile strength decreases considerably when a large amount of modified DCPA is added. The stress–strain curve shows that the presence of a large amount of hinokitiol in hydrogel membranes results in considerably improved deformation and toughness properties. Each group impregnated with hinokitiol exhibits obvious antibacterial capabilities. Furthermore, the addition of DCPA and impregnation with hinokitiol does not exert cytotoxicity on cells in vitro, indicating that the designed amount of DCPA and hinokitiol in this study is appropriate. After a 14-day cell culture, the hydrogel membrane still maintains a good shape because the cells adhere and proliferate well, thus delaying degradation. In addition, the hydrogel containing a small amount of DCPA has the best cell mineralization effect. The developed hydrogel has a certain degree of flexibility, degradability, and bifunctionality and is superficial. It can be used in guided tissue regeneration in clinical surgery.

## 1. Introduction

In periodontal restoration based on the concept of tissue regeneration, how to achieve regenerative membrane versatility has elicited extensive attention from many researchers [1,2,3,4]. Extant research mainly used different types of composite materials and adjusted different molecular structures or components to develop periodontal regeneration membranes for guided tissue regeneration (GTR) surgery. Previous studies have also used various strategies to meet the main requirements of absorbable regeneration membranes, such as durable mechanical properties, a sufficient degradation rate, and appropriate antibacterial properties that can still retain biocompatibility and osteoconductivity. Generally, different crosslinking technologies are utilized in order to improve the mechanical and degradation properties of hydrogel membranes. The addition of antibacterial factors or biologically active inorganic calcium phosphate endows periodontal regeneration membranes with antibacterial and biological activity or osteoconduction/osteoinductive properties [1,2].

In this study, gelatin and hyaluronic acid were selected as the main materials for the preparation of absorbable hydrogel regeneration membranes. The material structure design is based on the fact that gelatin is less immunogenic than collagen because it has an Arg-Gly-Asp (RGD) sequence, which is suitable for cell attachment and proliferation; the sequence also maintains the physiological functions of cells, and their degradation products can be absorbed and metabolized by the human body without causing tissue toxicity [5,6]. Similar to gelatin, hyaluronic acid has good biocompatibility and hydrophilicity, unique viscoelasticity, and low immunogenicity, and can regulate cell adhesion, proliferation, and differentiation; thus, it is widely used in biomedical devices for clinical applications, such as artificial joint synovial fluid, ophthalmic vitreous body, and wound surface regeneration used to treat osteoarthritis [7,8,9,10]. Gelatin and hyaluronic acid can undergo a crosslinking reaction through carbodiimide. Through the crosslinking reaction, gelatin can form a compact network structure, improve its mechanical properties, stabilize the structure of the hydrogel regeneration membrane, and slow down degradation. Furthermore, the addition of dicalcium phosphate anhydrous (DCPA) can easily release calcium ions in the hydrogel regeneration membrane to form a physically crosslinked egg box structure with gelatin. According to previous studies, DCPA is a bone resorption material and thus has good biocompatibility. As an additive in composites, DCPA improves absorption in bone tissues, has high osteoconductivity, and is suitable for bone regeneration [11,12]. An antibacterial factor can also be added. The natural ingredient hinokitiol can be isolated from Taiwan’s cypress plant. Hinokitiol can inhibit oral bacteria and is minimally toxic to normal oral cells. It has been used in toothpaste and oral care gel to improve oral lichen planus and bad breath [13,14,15,16,17,18,19,20].

In this study, gelatin and hyaluronic acid are adopted as the main structures, and *N*-(3-dimethylaminopropyl)-*N*’-ethylcarbodiimide hydrochloride (EDC) is used as a crosslinker to stabilize the mechanical properties of gelatin–hyaluronic-acid-based hydrogels. Hinokitiol is utilized as an antibacterial agent to reduce the risk of infection during periodontal tissue healing. In addition, DCPA particles are added to promote bone tissue regeneration and bone mineralization. The objective is to develop a dual-function hydrogel regeneration membrane with non-cytotoxic, antibacterial, and absorbable properties to promote soft and bone tissue regeneration.

## 2. Results

### 2.1. Reaction Mechanisms between the Hydrogels and Additives

**Figure 1:**
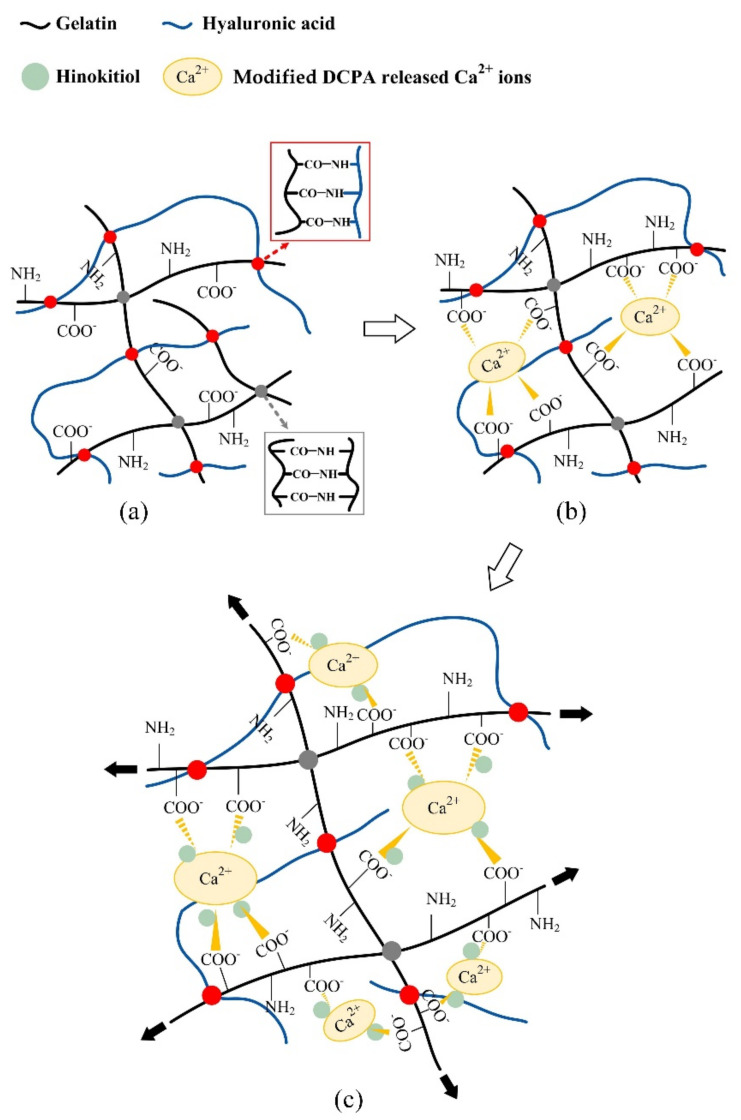
Schematic of a hydrogel composed of gelatin and hyaluronic acid without additives (**a**). Membranes with added modified DCPA show that the Ca^2+^ released by modified DCPA forms a physical bond with the carboxylic acid on the gelatin molecular chain, thereby inducing an ion–dipole bond between gelatin molecules (**b**). Adding a modified DCPA membrane and impregnating it with hinokitiol reduces the physical ion–dipole binding force between the gelatin molecules and allows for easy deformation because the long main chain of hyaluronic acid slides under tension, thereby reducing the elastic modulus of the hydrogel membrane (**c**).

The design concept of the GTR bifunctional hydrogel membrane developed by adding modified DCPA and impregnated with hinokitiol is shown in Figure 1. The network structure of gelatin and hyaluronic acid is mainly crosslinked by EDC. The main function of EDC is to activate the carboxylic acid residues of Asp and Glu in the RGD sequence of the gelatin structure, convert them into active *O*-acylisourea, and use the free amine of lysine to react with the carboxylic acid residues for the rearrangement of *O*-acylisourea into stable *N*-acylurea. The acid conducts a nucleophilic attack in order to form an amide bond [21]. The crosslinking of hyaluronic acid and EDC occurs through the initial formation of *O*-acylisourea on the polysaccharide, which reacts with adjacent carboxyl groups to form intermolecular crosslinks [22,23] (Figure 1a). Adding modified DCPA, a kind of absorbable bioceramic, quickly releases a large amount of divalent Ca^2+^ cations in contact with the liquid that penetrates into the hydrogel structure. The Ca^2+^ ion further promotes the network structure of gelatin, thus blocking the gelatin interaction with hyaluronic acid. Hyaluronic acid is an anionic non-sulfated glycosaminoglycan with a macromolecule backbone. The cation of Ca^2+^ forms a physical bond with the carboxylic acid group on the gelatin molecular chain. Therefore, with the addition of modified DCPA, the ion–dipole physical bond between Ca^2+^ and gelatin increases [24] (Figure 1b). However, hinokitiol with a hydroxyl and an isopropyl group leads to a large dipole moment [25,26]. Once hinokitiol is distributed in the hydrogel membrane, its hydrophobicity tends to cap the surface of hyaluronic acid instead of gelatin, and it interacts with Ca^2+^ at the same time, thereby reducing the network structure of the ion–dipole physical bond between Ca^2+^ and gelatin (Figure 1c).

### 2.2. Physiochemical Properties of Different Hydrogel Membranes

#### 2.2.1. Fourier Transform Infrared (FTIR)

**Figure 2:**
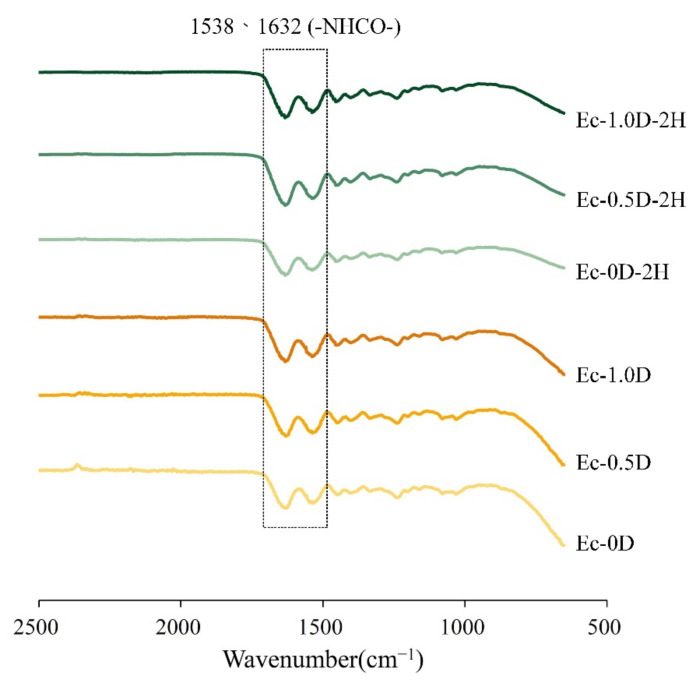
FTIR spectra of gelatin and hyaluronic acid crosslinked with EDC and loaded with hinokitiol/modified DCPA.

The FTIR spectra of the regenerated hydrogel membrane are shown in Figure 2. The functional groups of amide formed by the crosslinking reaction were observed at wavelengths of 1538 and 1632 cm^−1^ [27]. This observation shows that the crosslinking of hyaluronic acid and gelatin occurred through EDC, and that the additions of modified DCPA or hinokitiol to the hydrogels did not affect the crosslinking reaction.

#### 2.2.2. Strength Measurements

**Figure 3:**
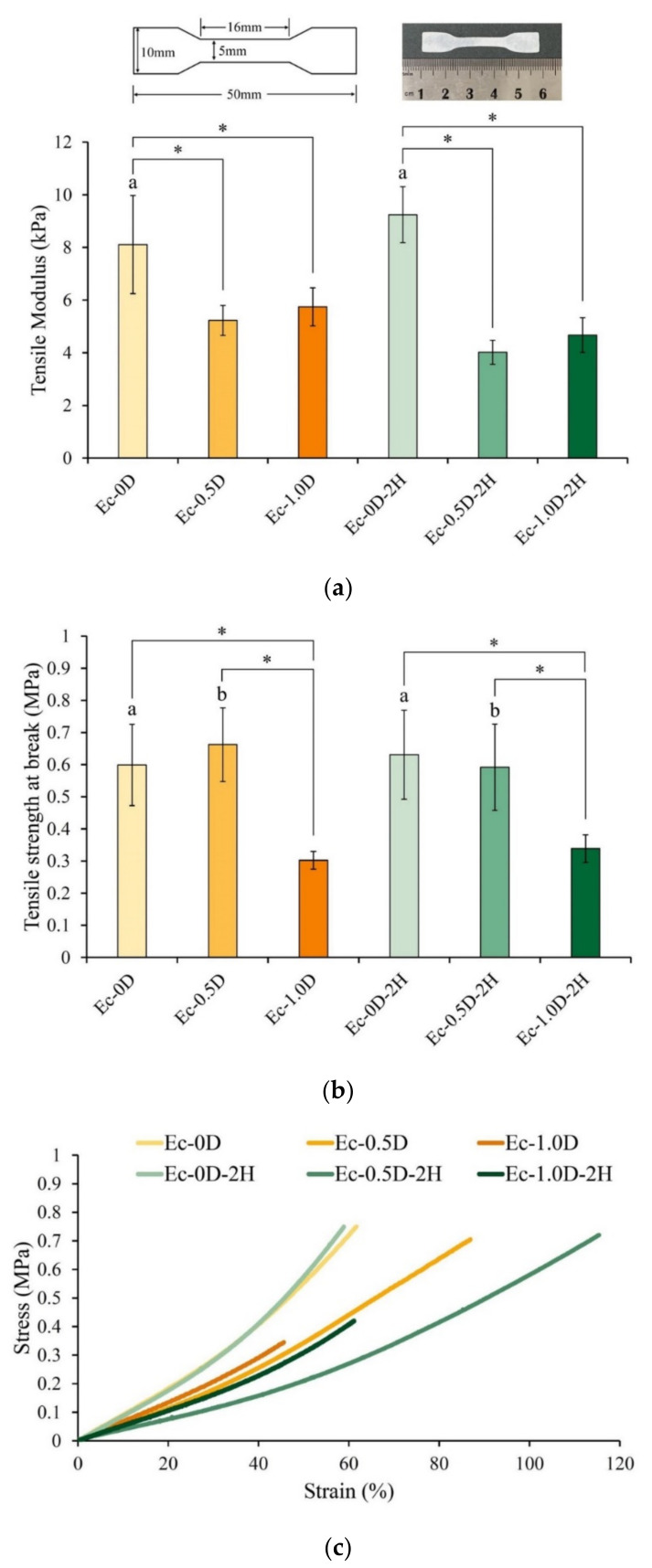
Measured modulus (**a**), ultimate strength at break (**b**), and stress–strain curves (**c**) of different hydrogel membranes after tensile testing (*n* = 10, the symbol * indicates that each group after one-way ANOVA shows *p* < 0.05, and the same alphabet indicates that each experimental group after *t*-Test shows *p* > 0.05).

The tensile results shown in Figure 3a indicate that a significant modulus difference existed between the Ec-0D and Ec-0.5D groups (*p* < 0.05). After adding modified DCPA to the hydrogel, the modulus significantly decreased. The modulus of Ec-1.0D with more modified DCPA was not significantly different from that of Ec-0.5D (*p* > 0.05), but the toughness of the membrane was improved. No significant difference in modulus was observed between the control of Ec-0D and the hinokitiol group of Ec-0D-2H (*p* > 0.05).

The ultimate breaking strength results showed that the breaking strength of the membranes containing more modified DCPA in the Ec-1.0D and Ec-1.0D-2H groups was significantly reduced (Figure 3b). Adding more modified DCPA reduced the tensile strength of the hydrogel properties. However, a comparison of the Ec-0D and Ec-0.5D groups with the Ec-0D-2H and Ec-0.5D-2H groups showed that no significant difference existed in the tensile strengths of the membranes containing hinokitiol (*p* > 0.05). Notably, the membrane with hinokitiol produced a stress–strain curve with increasing strain (Figure 3c), thereby increasing the toughness of the hydrogels with hinokitiol. We speculate that the reaction between hinokitiol and Ca^2+^ cation exerted a stronger polar effect than gelatin did. The decrease in the intermolecular force of gelatin was accompanied with the enhancement of the lubricating capability of hyaluronic acid.

#### 2.2.3. Topographies and Fracture Surfaces

**Figure 4:**
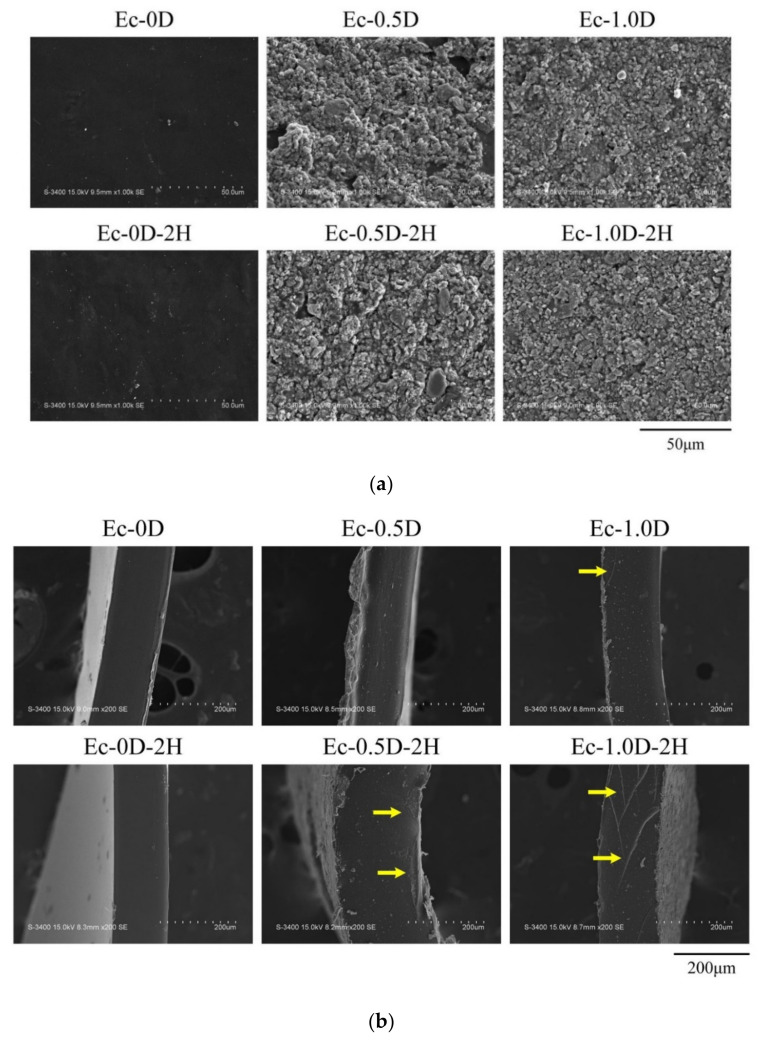
Surface images of different hydrogel membranes of EDC crosslinked without modified DCPA (Ec-0D) and with modified DCPA (Ec-0.5D and Ec-1.0D), and hinokitiol-impregnated hydrogels Ec-0D-2H, Ec-0.5D-2H, and Ec-1.0D-2H (**a**) and fracture surfaces of the hydrogel membranes after tensile testing (**b**) (the yellow arrowhead represents the toughness slips of the ductile failure of hydrogels through the particles).

The surface morphology of each group of hydrogel membranes is shown in Figure 4a. The morphology of the hydrogel membranes without the addition of modified DCPA was smooth, whereas the addition of modified DCPA membranes made the surface appear rough. The fractures of the membranes after the tensile test are shown in Figure 4b. The surface had a smooth cross section characterized by the brittle failure of the polymers occurring in groups Ec-0D and Ec-0D-2H. After adding modified DCPA to the Ec-1.0D group, fine modified DCPA particles were observed on the broken surface, together with shear lips at the outer surface, indicating the ductile failure of the hydrogels. This ductile failure was also evident in the Ec-0.5D-2H and Ec-1.0D-2H groups of hydrogels impregnated with hinokitiol.

#### 2.2.4. DSC and TGA Analysis

**Figure 5:**
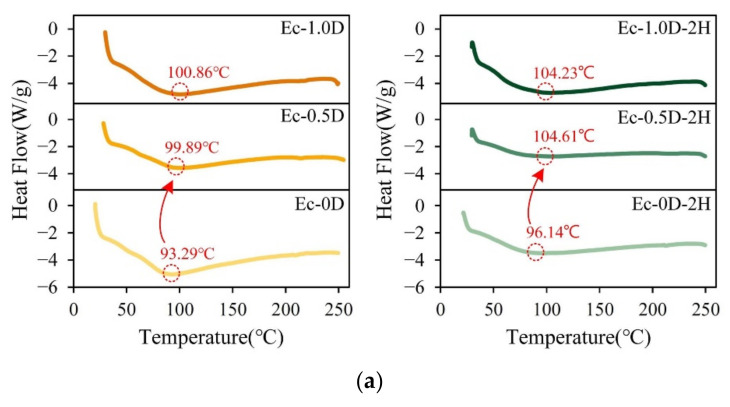

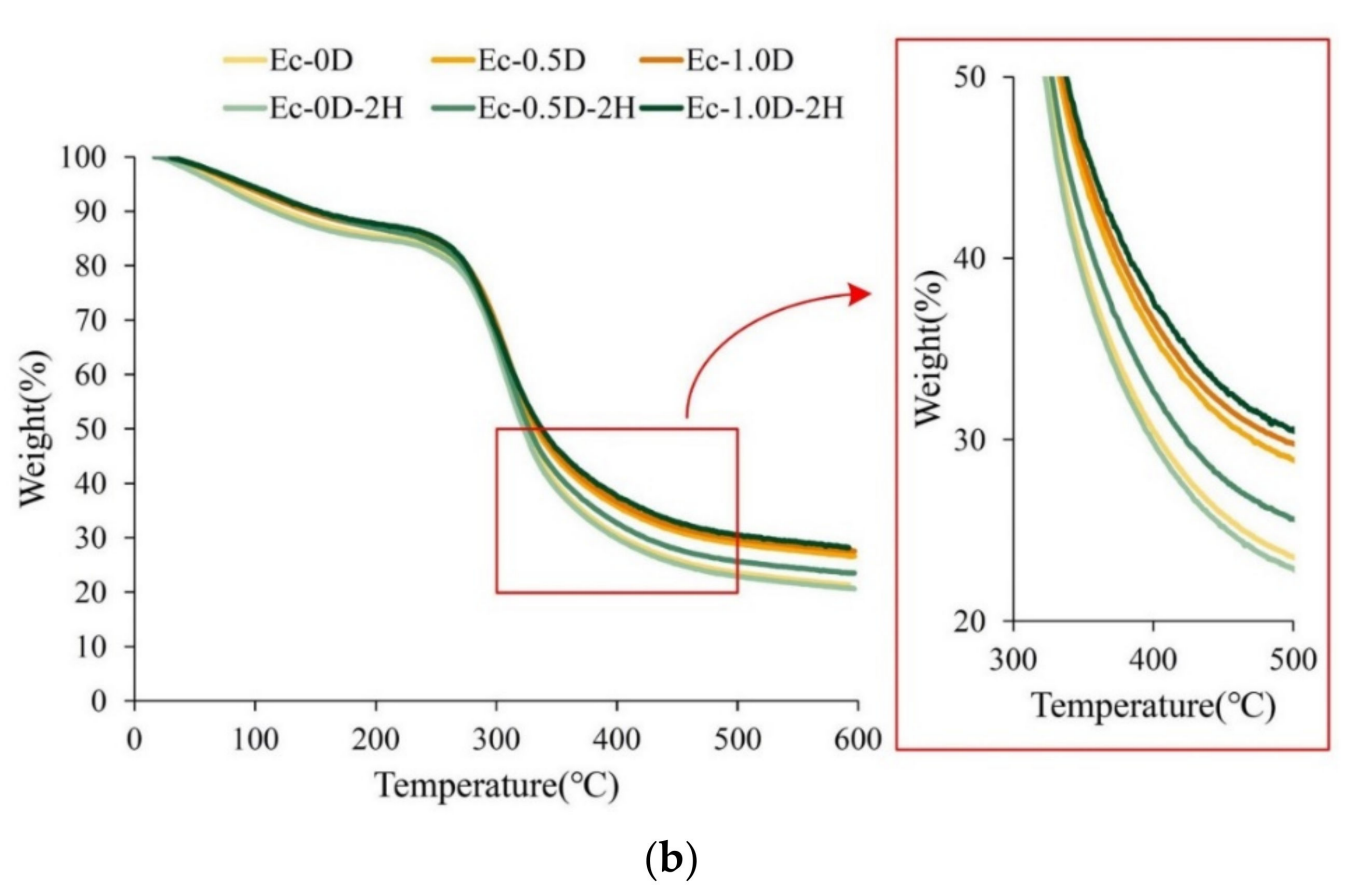
DSC analysis (**a**) and TGA (**b**) of different hydrogel membranes.

The DSC analysis results of the hydrogel membranes are shown in Figure 5a. Hydrogels usually contain some water, at a percentage that is usually 10–15%. Therefore, in addition to the water effect, the characteristic endothermic peaks shown in Figure 5a may be mainly due to physical bond interactions. Therefore, the degree of denaturation measured by DSC is a measure of the fraction of gelatin chains in gelatin–hyaluronic acid in the molecular structure. When the content of modified DCPA in the hydrogels increased, the denaturation temperature (Td) increased significantly. The Td of the control group (Ec-0D) and the membrane containing modified DCPA (Ec-1.0D) increased significantly from 93.29 °C to 100.86 °C. The results of Td for the groups of hydrogels impregnated with hinokitiol confirmed again that when the content of modified DCPA in the hydrogel increased, the temperature of Td also increased significantly. The result also reveals that the change in Td was not related to the hydrogel with hinokitiol. This phenomenon occurred because the Ca^2+^ cations released by the modified DCPA blocked the structure of gelatin and increased the Td of the hydrogel.

The weight change caused by the thermal degradation of the hydrogels was mainly observed through TGA (Figure 5b). The degradation in the curve could be divided into two stages. In the first stage, the weight of the membranes decreased to between 50 °C and 220 °C, mainly due to the evaporation of water inside the hydrogel. The second degradation stage started from 240 °C due to the amide decomposition upon pyrolysis. An observation of the two main groups showed that the proportion of thermal degradation decreased as the amount of modified DCPA increased in the membranes. This phenomenon verifies once again that adding modified DCPA to the hydrogel stabilized the structure.

#### 2.2.5. Evaluations of the Fixation Index, Swelling Ratio, and Degradation

**Figure 6:**
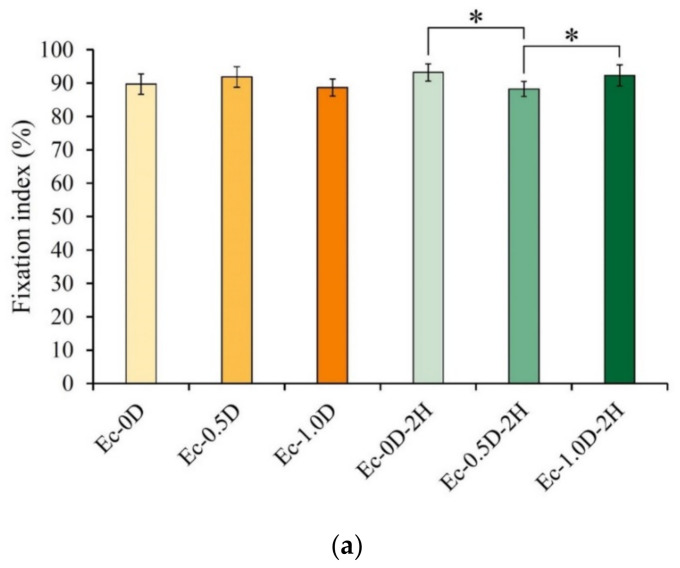

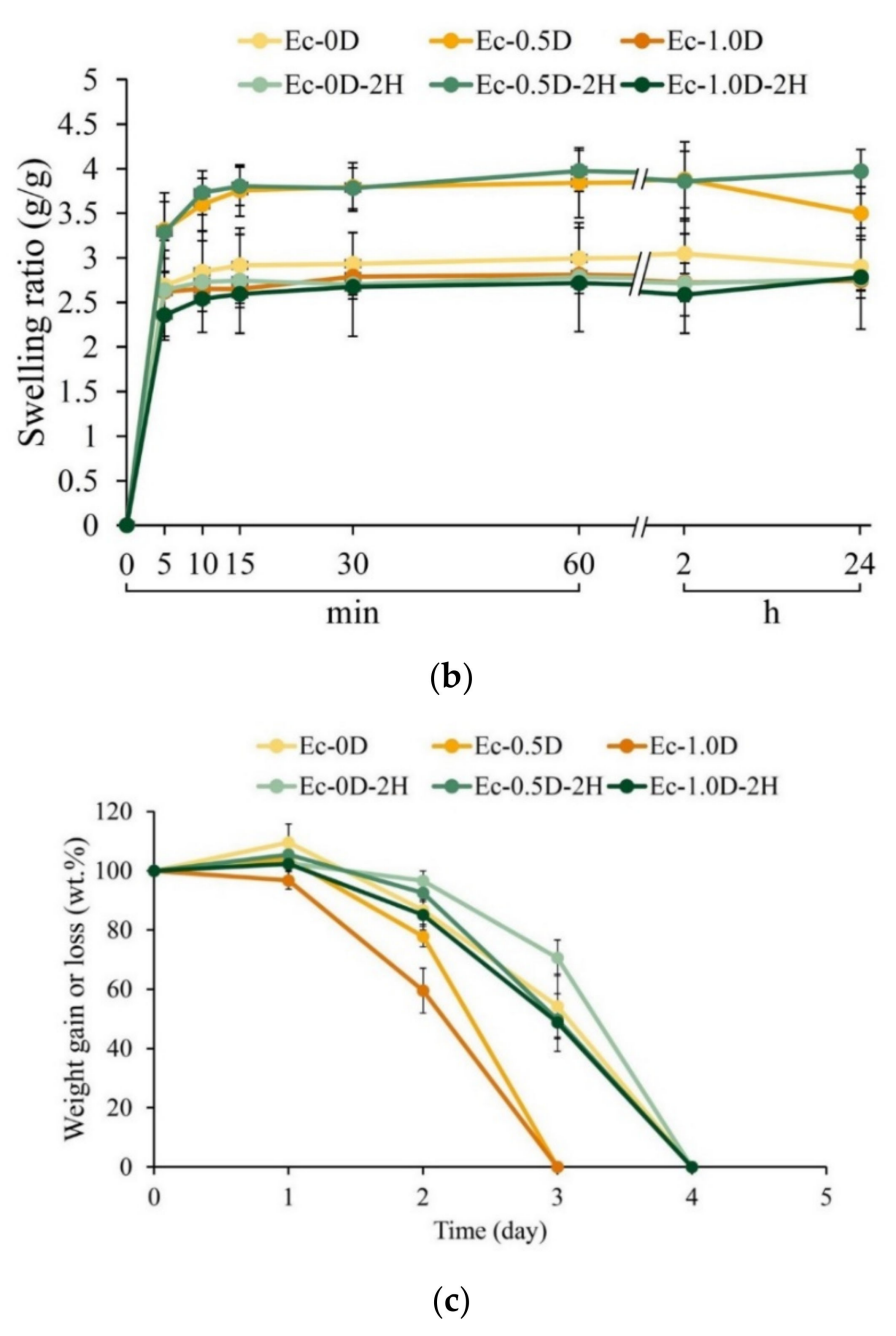
Fixation index (%) reflecting the degree of crosslinking of Ec-0D, Ec-0.5D, Ec-1.0D, Ec-0D-2H, Ec-0.5D-2H, and Ec-1.0D-2H (*n* = 3, the symbol * indicates that each group shows *p* < 0.05) (**a**). Swelling rates of different hydrogel membranes after 24 h of soaking (*n* = 10) (**b**) and weight changes in different hydrogel membranes during immersion in PBS (*n* = 6) (**c**).

The fixation index results for the different groups are shown in Figure 6a. In the hydrogel without hinokitiol, regardless of whether modified DCPA was added or not, no significant difference was observed between the groups (*p* > 0.05). The fixation index between the groups was significantly different (*p* < 0.05) compared with the index for the membrane containing hinokitiol. Each group’s fixation index was higher than 88%, indicating that gelatin and hyaluronic acid had good crosslinking through EDC and that the addition of modified DCPA had no obvious effect on the crosslinking reaction.

The weight change in the hydrogel membranes within 24 h after immersion in PBS is shown in Figure 6b. Each group of hydrogels quickly reached water saturation after 10 min of soaking and then achieved a plateau. A comparison of the weight change in the hydrogel until it was completely degraded is shown in Figure 6c. When immersed in PBS, only the Ec-1.0D group had a slight weight loss after 1 day of immersion; afterward, the measured weight of the hydrogel decreased significantly over time. The Ec-0.5D and Ec-1.0D groups were completely degraded on day 3 of immersion. All other groups, especially the hinokitiol group, continued to absorb water slowly after soaking for 1 day. After 2 days of soaking, the weight of these hydrogels decreased significantly, and complete degradation was delayed to the 4th day.

#### 2.2.6. Antibacterial Properties of Hydrogel Membranes with Hinokitiol

**Figure 7:**
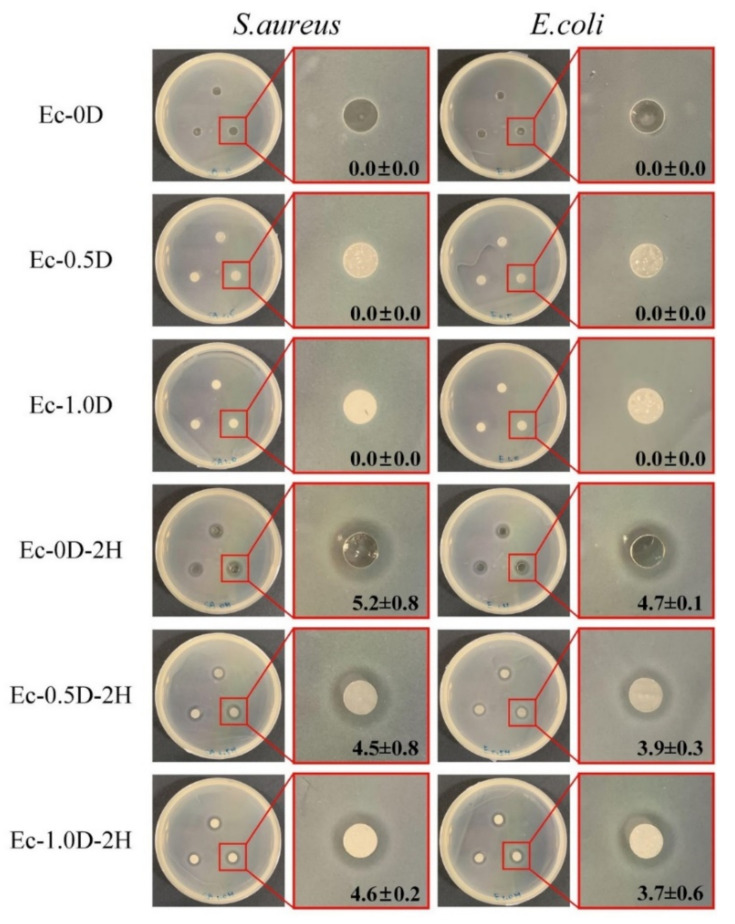
Qualitative images of the antibacterial properties of hydrogel membranes impregnated with different concentrations of hinokitiol for *S. aureus* and *E. coli* (*n* = 3).

The qualitative results of the antibacterial performance of the hydrogel membrane are shown in Figure 7. The group with modified DCPA but no hinokitiol had no inhibition zone. The hinokitiol impregnation group had obvious inhibition zones against *S. aureus* and *E. coli*, indicating that the designed concentration of hydrogel had good antibacterial capability.

### 2.3. Viability of Fibroblasts and Mineralization of Osteoprogenitor Cell

#### 2.3.1. Quantitative and Qualitative Evaluations of Cytotoxicity

**Figure 8:**
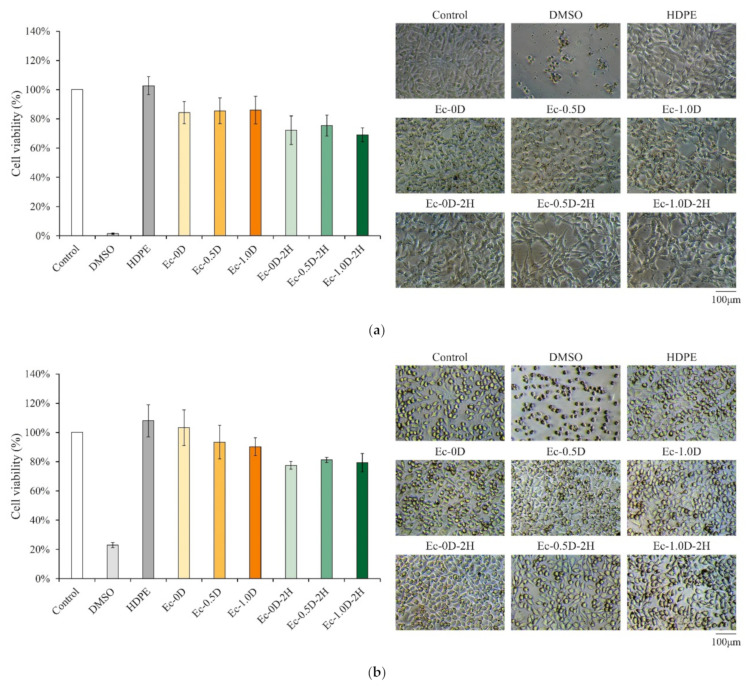
Quantitative (left, *n* = 6) and qualitative (right) cell images of the cytotoxicity of NIH-3T3 (**a**) and L929 (**b**) fibroblasts through different hydrogel membrane extracts (DMSO for positive control and HDPE for negative control).

After cell culturing for 24 h, fibroblastic NIH-3T3 (Figure 8a) and L929 (Figure 8b) cell viabilities were examined using extracts of hydrogel membranes with modified DCPA or hinokitiol impregnation. The cell viability of each group was greater than 70% relative to that of the control group, indicating that the hydrogel membrane did not affect cell activity nor did it cause obvious cytotoxicity. This result confirms that all of the components of the developed hydrogels in this study have good biocompatibility.

Figure 8a,b present optical images of NIH-3T3 and L929 cells cultured in the different extracts of hydrogel groups for 24 h. The images show that the cell type is healthy and spindle-shaped, thereby confirming that all experimental hydrogels were sterilized and did not cause adverse effects.

#### 2.3.2. Proliferation of the Hydrogels in Contact with Cells

**Figure 9:**
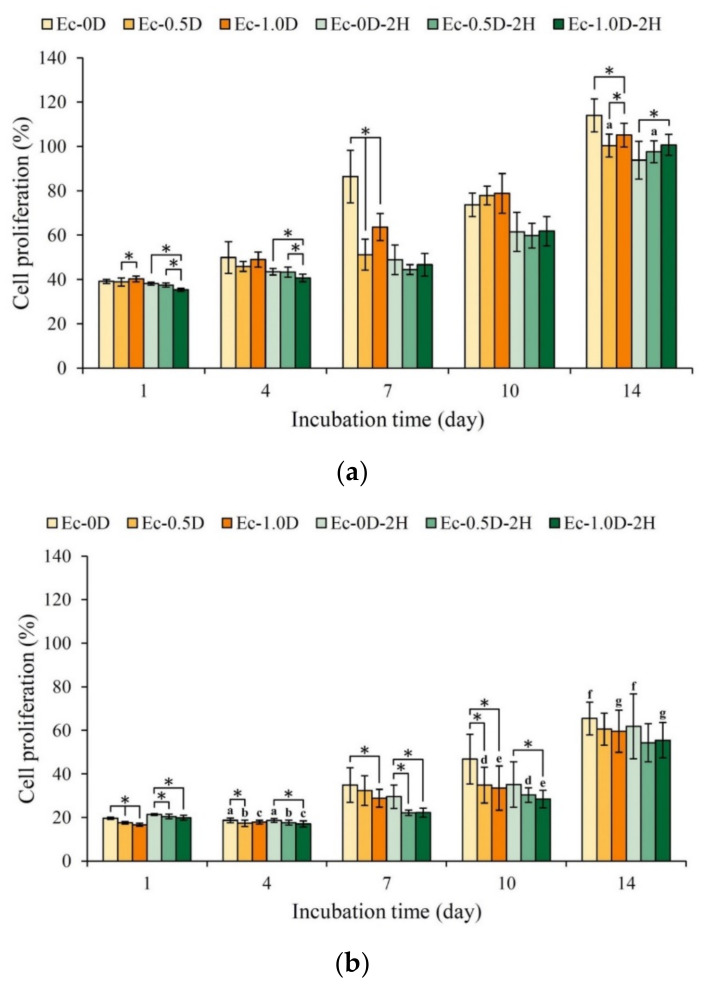
Proliferation of the hydrogel regenerated membrane in contact with NIH-3T3 (**a**) and L929 (**b**) cells for 1, 4, 7, 10, and 14 days (the symbol * indicates that each group shows *p* < 0.05, and the same alphabet indicates that the experimental group after *t*-test shows *p* > 0.05).

The NIH-3T3 fibroblasts were directly cultured and attached to the surfaces of different hydrogel membranes. As shown in Figure 9a, the hydrogels without hinokitiol had good viability on the 7th day of culture. Compared with the control Ec-0D group, the expression of cell adhesion and proliferation in the other groups was slightly worse. Figure 9b shows the cell viability of different hydrogel membranes attached to L929 cells. The expression of cell viability in each group exhibited the same trend as NIH-3T3, but the proliferation capability of L929 cells on the cell membrane was weaker than that of NIH-3T3.

**Figure 10:**
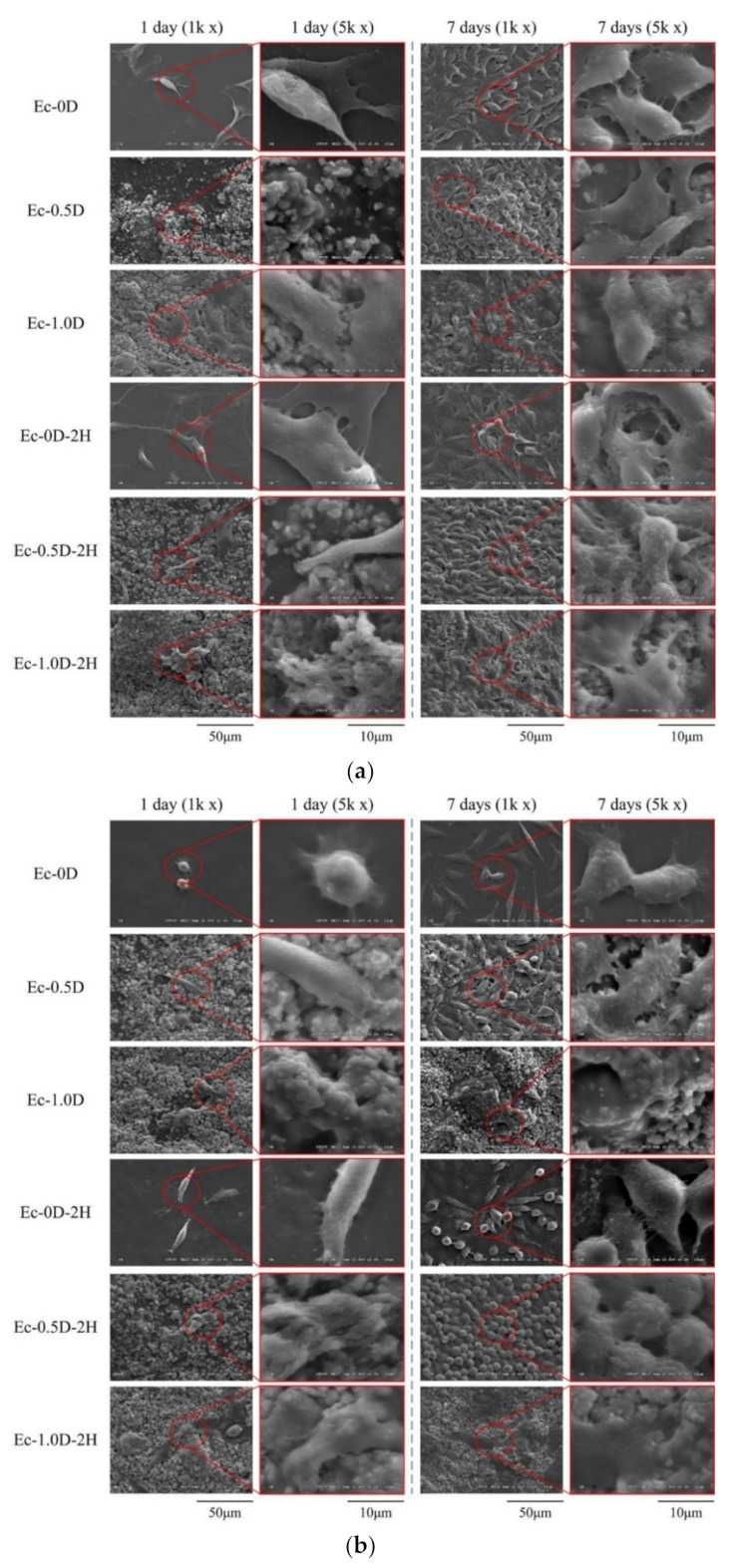
Images of cell attachment on the hydrogel membrane in contact with NIH-3T3 (**a**) and L29 (**b**) cells for 1 day and 7 days.

The morphologies of NIH-3T3 cells attached to the hydrogel membranes and cultured for 1 day and 7 days are shown in Figure 10a. When NIH-3T3 was cultured for 1 day, only a few cells adhered to the surface. However, after 7 days of culturing, multiple layers of cells attached and accumulated, indicating that NIH-3T3 was in a good growth state on all surfaces of the membrane. The cells were completely covered on the surface of the hydrogel membranes, revealing that the hydrogel membrane with cultured cells could maintain a better structure than the hydrogel membrane without cells; the cells prevented the hydrogel from swelling and degrading after soaking. The results also showed that the surface of the hydrogel containing modified DCPA was rough compared with the smooth surface without modified DCPA, which allowed the NIH-3T3 cells to adhere well initially. The morphological observation results of the L929 cells attached to the different hydrogel membranes for 1 day and 7 days were similar to those of NIH-3T3, but the qualitative and quantitative results were consistent (Figure 10b). Similar results are shown in Figure 9. Compared with the NIH-3T3 cells, the L929 cells proliferated slowly on the surface of the hydrogel membrane, and the surface was not filled up, even after 7 days of culture.

#### 2.3.3. Mineralization of the Hydrogels in Contact with D1 Cell

**Figure 11:**
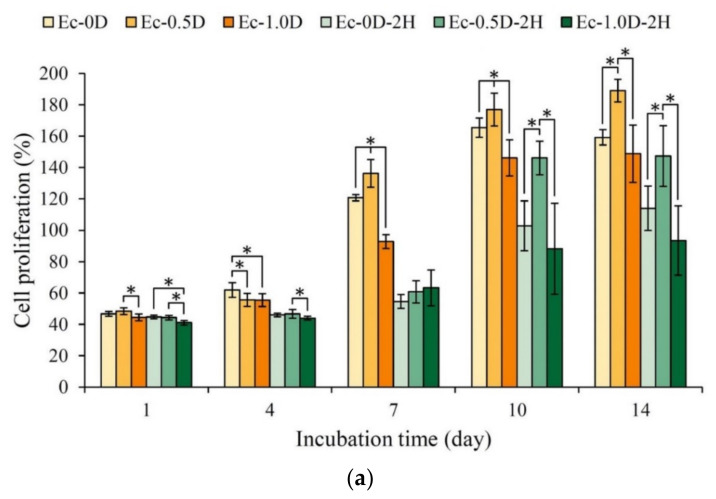

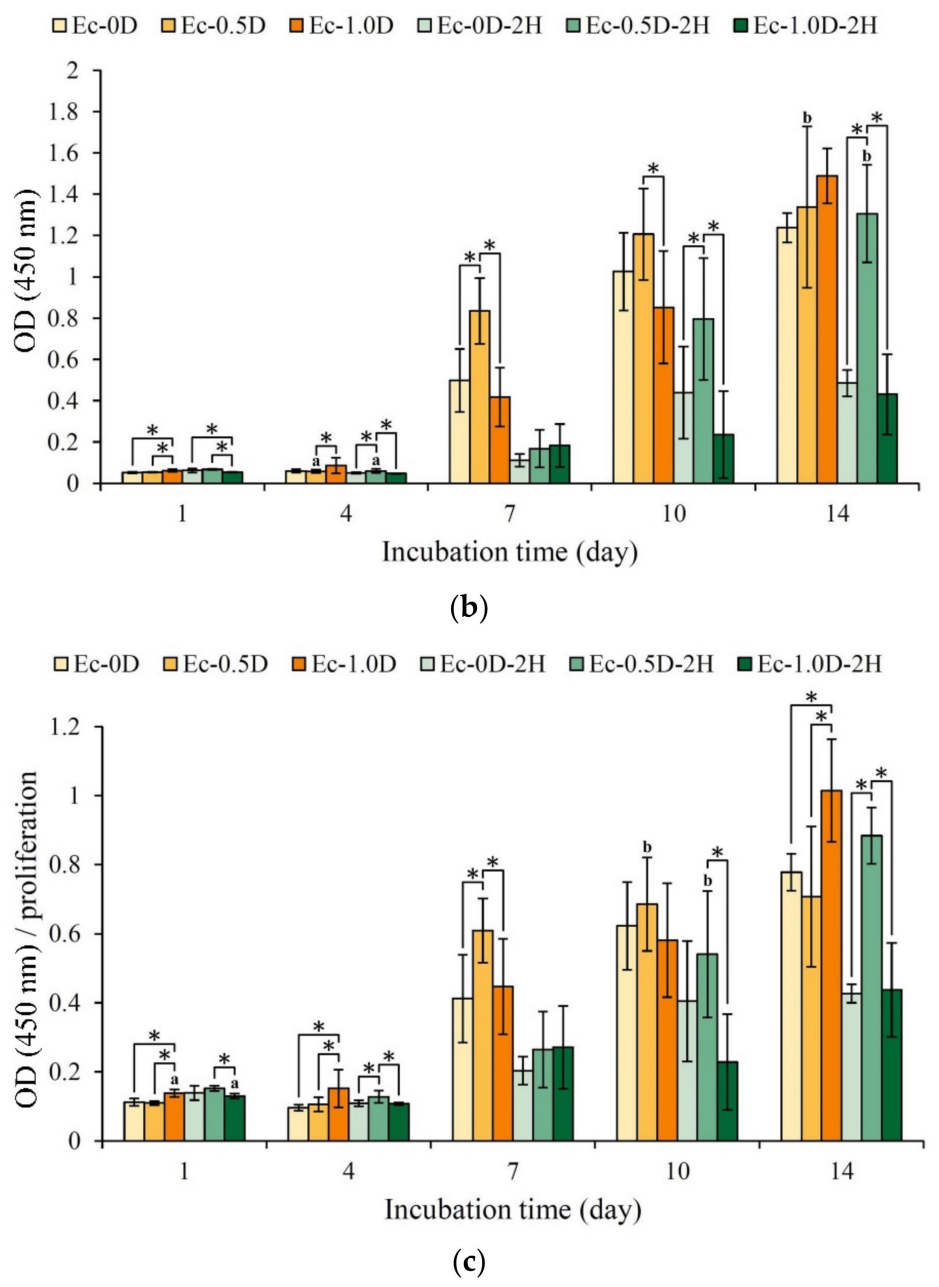
Cell proliferation of hydrogel membranes in contact with D1 cells for 1, 4, 7, 10, and 14 days (**a**). optical density OD_450_ of total cell ALP activity (**b**) and OD_450_/proliferation indicates ALP semi-quantitation (**c**) (the symbol * indicates that each group shows *p* < 0.05, and the same alphabet indicates that the experimental group after *t*-test shows *p* > 0.05).

The proliferation results of the hydrogel membranes cultured with osteoprogenitor cells D1 for 1–14 days are shown in Figure 11a. After D1 was cultured on the surfaces for 7 days, the group with the addition of modified DCPA, namely, Ec-0.5D, had the greatest proliferation effect. The cell proliferation capability in all of the hydrogels impregnated with hinokitiol was significantly slowed down, possibly because hinokitiol has an antibacterial effect that inhibits cell proliferation [26,28]. The ALP activity produced by the D1 cells after membrane contact culture is shown in Figure 11b. No obvious ALP production was observed in the groups with impregnated hinokitiol on the 7th day. As mentioned above, the Ec-0.5D group had the best ALP production capacity. The hinokitiol-impregnated group of Ec-0.5D-2H showed obvious ALP production on the 10th day. Given that the amount of ALP produced is also related to the number of cells, a semi-quantitative analysis of ALP could be performed (Figure 11c). The results showed that groups Ec-0.5D-2H and Ec-0.5D with/without hinokitiol had a good capability to secrete ALP.

**Figure 12:**
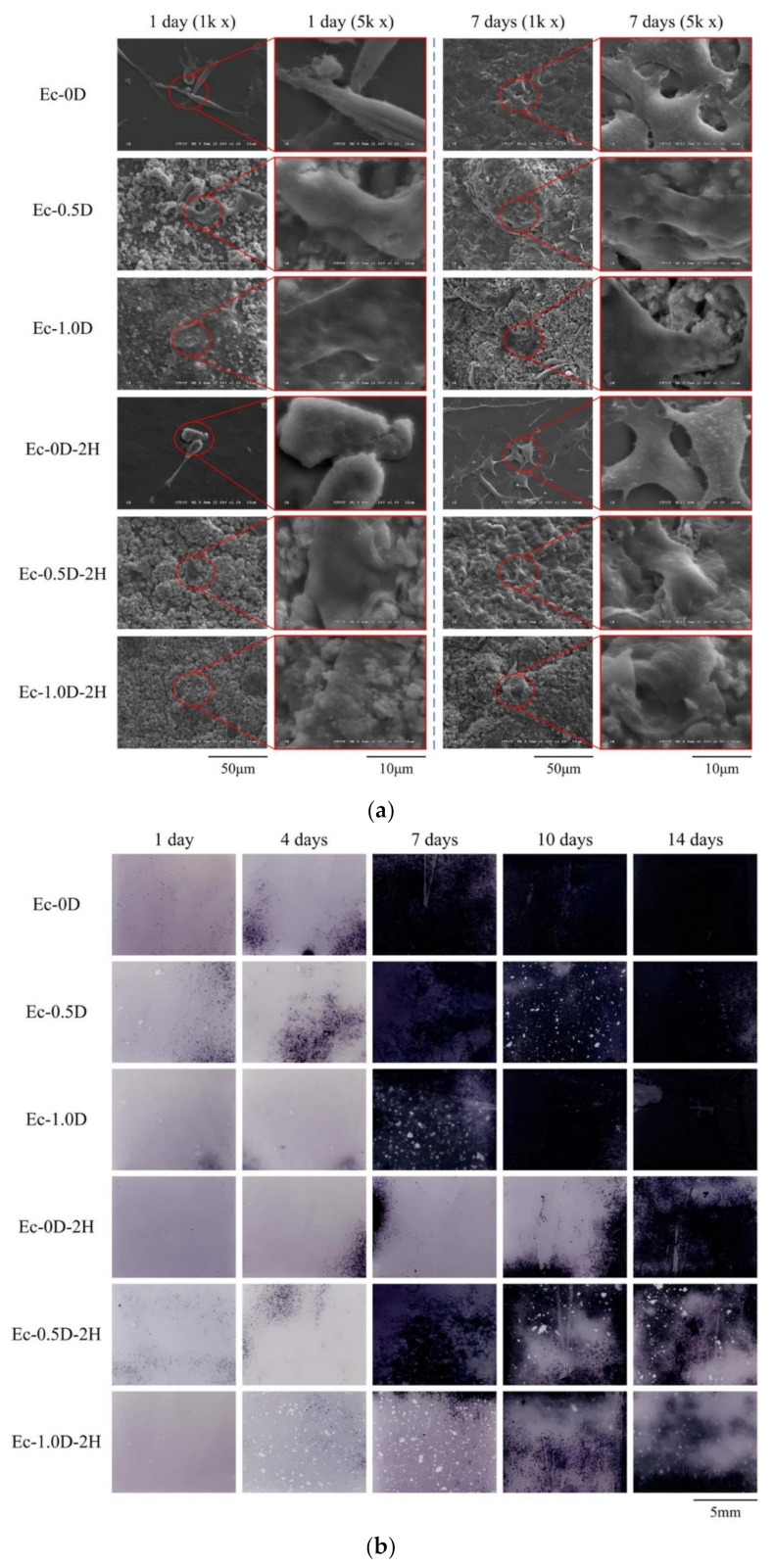
Images of cell attachment on the hydrogel membranes in contact with D1 cells for 1 day and 7 days (**a**) and ALP staining of the hydrogel membranes in contact with D1 cells and cultured for 1, 4, 7, 10, and 14 days (**b**).

The images of D1 cells attaching and proliferating on the surface of the hydrogel membrane for 1 day and 7 days are shown in Figure 12a. The result of osteoprogenitor D1 cell attachment is similar to that of fibroblasts. The results of ALP staining on the surface of the hydrogel membrane cultured with D1 cells for 1–14 days are shown in Figure 12b. No obvious ALP staining on the membrane surfaces was observed after culturing for 1 day in all of the groups. After 7 days of D1 cell culturing, the hinokitiol-free hydrogel groups showed obvious ALP staining, whereas the hinokitiol-impregnated hydrogels did not show obvious staining until after 10 days of culturing. The ALP staining results confirmed that the hinokitiol-impregnated group delayed the secretion of ALP in D1 mineralization during cell proliferation and differentiation.

## 3. Discussion

Given their high biocompatibility and sufficient affinity, Ca^2+^ ions are preferred for the gelation of gelatin because the divalent cation Ca^2+^ has strong affinities for hydrogen bonds, thus making the cross-linkage similar to that between oxygen or nitrogen atoms of the RGD sequence in gelatin and the divalent cation (Figure 1). Accordingly, in the stretching process of adding a small amount of modified DCPA to the Ec-0.5D group for the tensile test, part of the Ca^2+^ ions released by the particles formed physical bonds with the carboxylic acid or amine groups on the gelatin molecular chain [24]. Thus, the Ec-0.5D group could maintain its tensile breaking strength, which is compatible with that of the control Ec-0D group (Figure 3b). However, many Ec-1.0D groups of modified DCPA were added because many modified DCPA particles and gelatin molecules formed numerous cation–dipole physical bonding points, thus creating a complex network structure. On the contrary, when the developed hydrogel membrane was stretched, the molecular chain of gelatin was affected by the hydrogen dipole bond with cation Ca^2+^, resulting in a significant decrease in tensile strength (Figure 3b). The developed hydrogel groups impregnated with hinokitiol significantly increased the toughness of the regenerated hydrogels (Figure 3c). We speculate that the phenomenon may be due to the hydrophobicity of hinokitiol, which reduces the original physical cation–dipole bonding so that the structure can be maintained during the stretching process. The structure between the crosslinked segments of hyaluronic acid and gelatin, especially in an aqueous environment (Figure 6c), is protected by hydrophobic groups due to the impregnated hinokitiol, which can extend the structural stability of the hydrogel.

Research has shown that the RGD sequence can promote cell adhesion in tissue engineering applications [25,26]. Resorbable bioceramics are largely incorporated into biodegradable polymer blends used in bone tissue engineering applications [29]. In cell adhesion in this study, we observed that the hydrogel group added with a combination of resorbable bioceramics of modified DCPA and hinokitiol exerted no cytotoxicity on NIH-3T3 and L929 cells, and during long-term cell cultures, the cells that adhered to the surface of the hydrogel membrane prevented the hydrogel membrane from swelling after degradation in vitro (Figure 9). After the long-term culture of D1 cells in group Ec-0.5D, the group showed the best proliferation capability, and ALP production became obvious on the 7th day. The hydrogel was further combined with the hinokitiol group of Ec-0.5D-2H, and it showed an obvious proliferation capability and delayed ALP production after 10 days of culture. This phenomenon is speculated to be due to the release of hinokitiol during culture, which affects cell proliferation and ALP production [26]. According to studies, the use of osteogenic factors is not effective for bone tissue regeneration before eliminating bacterial infections and inflammatory symptoms; thus, bacterial infections and inflammatory symptoms must be controlled before assisting osteoregeneration [4,30,31].

This research is currently limited to in vitro testing. Although hinokitiol immersion on hydrogels delays cell proliferation and ALP production, hinokitiol can achieve a good antibacterial mechanism in the early stage of tissue regeneration. This study showed that D1 cells achieve good cell proliferation and ALP production only after the culture process is delayed to the 10th day. It confirmed that the addition of modified DCPA can still promote ossification. As is well known, one of the hallmarks of chronic wounds is the prolonged period of inflammation caused by the production of ROS and matrix-degrading enzymes (e.g., proteases and matrix metalloproteinases). Therefore, the most important mechanism of hyaluronic acid in tissue regeneration is its capability to function as a free radical scavenger in the process of tissue granulation formation [32]. Hinokitiol exerts a significant protective effect against epithelial surface inflammation by inhibiting the NF-κB pathway, which indicates that it could relieve inflammation [33]. Therefore, in order to prove the practicability of this research, the inflammation model must be verified in vivo through animal experiments in the future.

## 4. Materials and Methods

### 4.1. Raw Materials

The raw materials were gelatin (type B from bovine skin with an average molar mass of 40,000–50,000 g/mole; Sigma-Aldrich^®^, St. Louis, MO, USA), hyaluronic acid (molecular weight ranging within 8–10,000 kDa; Foodchemifa Co., Ltd., Tokyo, Japan), EDC (molecular weight of 191.70 g/mole; Sigma-Aldrich^®^, St. Louis, MO, USA), spherical micrometer-scale modified powder of DCPA (modified DCPA; REALBONE TECHNOLOGY Co., Ltd., Kaohsiung, Taiwan), and hinokitiol (purity of 99.9%; Sigma-Aldrich^®^, St. Louis, MO, USA). The modified DCPA powder had a particle distribution size ranging from 1 μm to 3 μm, and a powder with 98% purity was used.

### 4.2. Preparing Hydrogel Specimens

According to the literature and our preliminary research [27,34], gelatin (7 g) and hyaluronic acid (0.07 g) were mixed with 70 mL of a 50 vol.% alcohol solution. The mixture was heated to 55 °C and stirred for 30 min. Then, 0.5 g and 1.0 g of the modified DCPA powder were added, and the mixtures were continuously stirred for 30 min. Afterward, the colloidal hydrogels with and without (w/o) modified DCPA were placed on a stainless steel plate. The hydrogels were carefully controlled to form a 0.34 mm thick sample with a standard deviation of 10%. The sample was subjected to molding for 4 h and dehumidified at 25 °C for 24 h. Then, it was demolded, soaked in 1% EDC crosslinking agent, kept in a refrigerator at 4 °C for 24 h of crosslinking, and rinsed with deionized water three times. Afterward, EDC crosslinked hydrogel membranes (i.e., without modified DCPA (Ec-0D) and with modified DCPA (Ec-0.5D and Ec-1.0D)) were obtained. The hydrogel membranes were cut into dumbbell-shaped specimens for hinokitiol loading and mechanical tests. The detailed dimensions are shown in the figures presenting the results of the strength testing.

In this experiment, the solvent used to dissolve hinokitiol was phosphate-buffered saline (PBS). In brief, 0.002 g of hinokitiol was dissolved in 25 mL of PBS due to hinokitiol’s compatibility according to our previous study. The time for soaking each EDC crosslinked hydrogel sample in the prepared hinokitiol containing PBS was set to 15 min. Then, the specimens were dried at room temperature in order to obtain hinokitiol-impregnated hydrogels Ec-0D-2H, Ec-0.5D-2H, and Ec-1.0D-2H.

### 4.3. Functional Groups from Infrared Spectrum

Attenuated total reflectance Fourier transform infrared spectroscopy (Ni-colet 6700, Thermo Fisher Scientific, Waltham, MA, USA) was used to analyze the combined effects of hinokitiol and modified DCPA on the crosslinking hydrogel.

### 4.4. Tensile Measurements

Each group of hydrogel membranes was made into dumbbell-shaped test samples, the thickness of which was approximately 0.28–0.36 mm after PBS absorption. The detailed dimensions are shown in the figures presenting the results of the strength testing. A universal strength testing machine (HT-2402, Hongda Instrument Co., Ltd., Taichung, Taiwan) was used to measure the tensile strength at a rate of 2 mm/min and the strength curve from 0 N to the sample break. The tester read sample changed in tensile strength and elongation, and the tensile modulus was calculated using the formula below, where Δσ is stress (N/mm^2^: MPa) and Δε is strain (mm/mm).

(1) $$\mathrm{Tensile}\;\mathrm{modulus}\;\left(\mathrm{MPa}\right)=\frac{\Delta \sigma }{\Delta \varepsilon }$$

The tested membrane was plated with metal, and the surface and fracture morphologies of the hydrogels were observed using a scanning electron microscope (SEM; Hitachi S-3000N, Hitachi, Tokyo, Japan).

### 4.5. Characterization of the Hydrogels’ Thermo-Physical Properties

A modulated differential scanning calorimetry instrument (DSC; TA Instrument, New Castle, DE, USA) was used to heat the sample from room temperature to 300 °C at a heating rate of 10 °C/min. The enthalpy change in the sample during heating was measured. The sample was heated at a constant temperature rate to infer the properties of the sample. In addition, a thermogravimetric analysis instrument (TGA; TA Instrument, New Castle, DE, USA) was used to measure the residual weight change in the sample from room temperature 600 °C at a heating rate of 10 °C/min.

### 4.6. Hydrogel Fixation Indices of the Crosslinking

Given that the free amines of gelatin and hyaluronic acid react with EDC to form amide, the degree of crosslinking is proportional to the free amine fixation index of the hydrogel. An amount that is opposite to that of the residual amine can be used to evaluate the degree of crosslinking of the hydrogel. Ninhydrin (2,2-dihydroxy-1,3-indenedione) reagent (Sigma-Aldrich^®^, St. Louis, MO, USA) is an organic compound widely used for the detection of amino acids. In this study, ninhydrin was utilized for colorimetric determination in order to compare the semi-quantitative values of the residual functional groups of active amino acids exposed outside the backbone of gelatin and hyaluronic acid before (*activated amino*) *_fresh_* and after crosslinking through EDC (*activated amino*) *_residues_*. This reagent oxidatively decarboxylates amino acids to produce CO_2_, NH_3_, and aldehydes with one carbon fewer than the original amino acids. The reduced ninhydrin and NH_3_ form a blue–violet product; therefore, the more NH_3_ that is produced, the darker the color of the product. In accordance with the color changes produced by the reaction, the unreacted amino acid content can be calculated through regression at an optical density of 570 nm (OD_570_) via an enzyme-linked immunosorbent assay (ELISA) reader. First, 1 mg of each hydrogel with and without EDC crosslinking was immersed in deionized water for 1 h. Then, the ninhydrin reagent was added and the mixture was heated to 100 °C for 10 min in order to measure the OD value. The calculation formula where the fixed index is proportional to the degree of crosslinking is [35]:

(2) $$\mathrm{Fixation}\;\mathrm{index}\;\left(\%\right)=\frac{{\left(activated\;amino\right)}_{fresh}-{\left(activated\;amino\right)}_{residual}}{{\left(activated\;amino\right)}_{fresh}}\times 100\;\%$$

### 4.7. Solution Absorption of Hydrogels

The hydrogel membrane was immersed in PBS with and without hinokitiol. The varied weights of the samples that could be absorbed in the solution were measured. The dried hydrogel membrane samples were initially weighed (*W*_0_), and the weights (*W_t_*) after immersion in periods of 5 min, 10 min, 15 min, 30 min, 60 min, 2 h, and 24 h were measured. This measurement was performed after the samples were removed from PBS and the residues on the surfaces were wiped with saturated sponges. The equation for calculating the weight change in the hydrogel was as follows:

(3) Weight varying ratio (%)=Wt−W0W0 gg×100%

### 4.8. Degradation Rate of Hydrogels

The samples were pre-immersed in PBS for 24 h, weighed (*W_sat_*), and taken out. The residual weight (*W_residual_*) was measured every day until the samples were completely degraded. The excess water was wiped from the surface slightly and the residual weight (*W_residual_*) of each sample was measured until the sample was completely degraded. The calculation formula of the sample weight change is as follows:

(4) $$\mathrm{rate}\;\mathrm{of}\;\mathrm{weight}\;\mathrm{gain}\;\mathrm{or}\;\mathrm{loss}\;\mathrm{to}\;\mathrm{degradation}\;\left(\%\right)=\left[1-\frac{W_{sat}-W_{residual}}{W_{sat}}\right]\times 100\%$$

### 4.9. Evaluation of the Hydrogel Membrane with Antibacterial Properties

*Staphylococcus aureus* (*S. aureus*, ATCC number: 25923) and *Escherichia coli* (*E. coli*, ATCC number: 10798) grown in Luria–Bertani broth were used. Liquid broth (1.5%) was sterilized in an autoclave, balanced in a 50 °C water bath for 30 min, and poured into 10 cm petri dish to obtain solidified tryptic soy broth (TSB) agar. When OD_595_ nm = 0.2 was measured, 250 μL of the bacterial solution was aspirated, and the bacterial solution was placed on solidified TSB agar for culturing. Then, a circular sample with a diameter of 0.7 mm was placed on the surface of the solidified TSB agar and incubated at 37 °C for 24 h in order to measure the inhibition zone.

### 4.10. Viability of NIH-3T3 and L929 Fibroblasts Cultured in Sample Extracts

Commercial cell lines of NIH-3T3 and L929 were used to test the in vitro cytotoxicity of the synthetic hydrogel membranes. The used cell culture media were purchased from Gibco Thermo Fisher Scientific Inc., Waltham, MA, USA. The extraction ratio of the hydrogel membrane to the cell culture medium was set to 0.1 g/mL in accordance with the ISO 10993-5:2009 guideline for 24 h immersion. The culture medium for NIH-3T3 cells was Dulbecco’s modified Eagle’s medium (DMEM), which contains 10% bovine serum. The medium for L929 cells was modified minimum essential medium (MEMα) containing 10% horse serum.

For comparison, the viabilities of the NIH-3T3 and L929 cell control group (group cultured with medium only) were set to 100%. In accordance with the regular ISO 10993-5 guideline, 15% dimethyl sulfoxide (DMSO) was used as positive control for cytotoxicity. High-density polyethylene (HDPE) extract was utilized as negative control in order to ensure the validity of sample sterilization. The designated control group was the medium group, and the experimental groups contained the extracts of hydrogel membranes.

Cells with a cell density of 1 × 10^4^ were inoculated in a 96-well culture plate and were incubated for 24 h, the medium was aspirated, and the sample extract was added to the culture plate. After 24 h of culture, the cell viability was measured at OD_490_ nm by an ELISA reader. A commercially available tetrazolium salt (XTT assay, Biological Industries, Beit Haemek, Israel) was used. Cell morphological characteristics were observed under an optical microscope (IVM-3AFL, SAGE VISION Co., Ltd., New Taipei City, Taiwan).

### 4.11. Proliferation and Attachment of NIH3T3, L929 and D1 Cells on Hydrogels

Three types of cells, namely, NIH-3T3, L929, and D1, were used for cell attachment culture and observation. The medium used for the D1 cells was DMEM containing 10% standard fetal bovine serum. Fibroblasts were at a concentration of 1 × 10^5^ cells/cm^2^ seeded in 48-well plates, and were incubated for 1, 4, 7, 10, and 14 days. At different incubation times, the cells were cultured with the alamarBlue Proliferation Assay Kit (AbDSerotec Ins., Raleigh, NC, USA) for extended 4 h, and were measured at OD_570_ and OD_595_ nm with an absorbance plate reader (SPECTROstar Nano, BMG LABTECH, Ortenberg, Germany).

For a comparison of cell observations, the cells were washed with PBS for 1 day and 7 days after culture, and then dehydrated with 2.5% glutaraldehyde, paraformaldehyde, and different concentrations of alcohol. Afterward, the hydrogel with cells was gold plated, and the cell morphologies were observed via SEM.

### 4.12. Alkaline Phosphatase Activities and Staning

Alkaline phosphatase (ALP) testing was performed simultaneously with the same intervals as those in the cell proliferation tests. The production of ALP (early marker of osteogenesis) was measured as follows. The p-nitrophenyl phosphatase (Merck KGaA, Darmstadt, Germany) and TBS in the p-nitrophenyl phosphatase kit was added to 20 mL of sterile water and mixed well. After incubation, the cells were washed with PBS once and added to 1 mL of the prepared solution in order to incubate the cells for 30 min. Then, the absorbance of the ALP secreted by the cells was measured with an ELISA reader at OD_450_. ALP staining was performed using the SIGMAFAST™ BCIP^®^/NBT kit (Merck KGaA, Darmstadt, Germany) in accordance with the manufacturer’s instructions.

### 4.13. Statistical Analysis

ANOVA and the two-sample *t*-test in IBM SPSS Statistics Version 20 (IBM, Armonk, NY, USA) were applied to discuss and analyze the differences in pore size, porosity, degree of crosslinking, and water absorption. Statistical analysis was also performed to assess significant differences (*p* < 0.05).

## 5. Conclusions

Modified DCPA powder was added to gelatin and hyaluronic acid, crosslinked by EDC, and impregnated with the bacteriostatic factor hinokitiol to prepare a dual-function hydrogel membrane. The developed membrane has antibacterial properties and promotes bone tissue regeneration for GTR surgery. The conclusions of this study are summarized as follows:

(1) Adding modified DCPA powder combined with hinokitiol does not affect the crosslinking index of the hydrogels, and the further impregnation of the hydrogel membranes with hinokitiol can delay immersion degradation;
(2) Adding modified DCPA powder to the hydrogel group significantly reduces its modulus. However, because the cation Ca^2+^ forms a physical ion–dipole bond on the gelatin molecular chain, a small amount of addition during stretching cannot significantly reduce the tensile strength. When a large amount of modified DCPA is added, the tensile strength decreases significantly;
(3) In the contact culture with D1 cells, the group without hinokitiol exhibited obvious ALP production on the 7th day of culture, and the Ec-0.5D group with a small amount of modified DCPA had the best ALP production capability;
(4) The hydrogel membrane impregnated with hinokitiol (Ec-0.5D-2H) showed significant antibacterial activity against *S. aureus* and *E. coli*. In the cell viability test, the addition of modified DCPA powder and impregnation with hinokitiol had no adverse effects on NIH-3T3 and L929 cells. The Ec-0.5D-2H group may be the best hydrogel membrane candidate for guiding tissue regeneration in in vitro tests.

## Data Availability

Data are contained within the article.
